# Expression of CD4+CD25+CD127^Low^ regulatory T cells and cytokines in peripheral blood of patients with primary liver carcinoma

**DOI:** 10.7150/ijms.44088

**Published:** 2020-02-24

**Authors:** Wenchao Zhou, Jianxin Deng, Qianmei Chen, Ruiying Li, Xiaosong Xu, Yubin Guan, Wei Li, Xiaomin Xiong, Hongwei Li, Jianpei Li, Xiangsheng Cai

**Affiliations:** 1 Clinical laboratory, the First Affiliated Hospital of Guangdong Pharmaceutical University, Guangzhou 510080, China; 2 Department of Endocrinology, Shenzhen Second People's Hospital, The First Affiliated Hospital of Shenzhen University, Health Science Center of Shenzhen University, Shenzhen 518035, People's Republic of China.; 3 Clinical laboratory, Guangzhou Military Area Inspection Center, the General Hospital of Guangzhou Military Region, Guangzhou 510010, China; 4 Clinical laboratory, the Hospital of Dongguan Renkang, Dongguan 523952, China; 5 Institute of Biotherapy, Southern Medical University Guangzhou 510515, China; 6 State Key Laboratory of Oncology in South China, Collaborative Innovation Center for Cancer Medicine, Department of Clinical Laboratory Medicine, Sun Yat-Sen University Cancer Center, Guangzhou 510060, China

**Keywords:** CD4+CD25+CD127^low^ regulatory T cells, cytokines, flow cytometry, peripheral blood, primary hepatic carcinoma

## Abstract

**Objective:** To assess the clinical utility of the ratio of CD4+CD25+CD127^low^ regulatory T cells (Tregs) in subjects at high risk of HCC, investigate the relationship between the percentage of Tregs and the expression of transforming growth factor (TGF)-β1 and interleukin (IL)-10 in patients with hepatocellular carcinoma before and after treatment.

**Methods:** Peripheral venous blood was collected from patients with liver cancer before and after treatment. The proportion of CD4+CD25+CD127^low^ Tregs was detected by flow cytometry. The levels of TGF-β1 and IL-10 in serum were detected by enzyme-linked immunosorbent assay, and were compared with healthy subjects as a control group.

**Results:** The proportion of CD4+CD25+CD127^low^ to CD4+T lymphocytes in patients with hepatocellular carcinoma was significantly higher than that in healthy controls (*P<0.01*). The proportion of CD4+CD25+CD127^low^Tregs, whose AUC of ROC curve was 0.917, could effectively separate the HCC patients from the healthy subjects with a diagnostic sensitivity of 90%, specificity of 80%. The proportion of CD4+CD25+CD127^low^ to CD4+T lymphocytes and the levels of TGF-β1 and IL-10 in patients with hepatocellular carcinoma after the operation and chemotherapy were significantly lower than those before treatment (P<0.05).The proportion of CD4+CD25+CD127^low^Tregs was positively correlated with the concentrations of TGF-β1 and IL-10 before and after treatment of primary liver cancer (P<0.05).

**Conclusion:** CD4+CD25+CD127^low^Tregs may be a significant predictor of HCC biopsy outcome and play an inhibitory role on effector T cells by regulating cytokines.

## Introduction

The annual incidence of primary liver cancer ranks fifth among all malignant tumors in the world, and the mortality rate ranks third 1. Surgical treatment is the only hope for long-term survival of patients with liver cancer; however, most patients with liver cancer in China are diagnosed at an advanced stage, and they have lost the chance for successful radical surgical resection 2. CD4+CD25+ regulatory T cells (Tregs) are a subset of T lymphocytes with immunosuppressive properties that play an important role in the regulation of peripheral immune responses^3^. CD127 is a recently discovered antigen associated with Tregs that is weakly expressed on Tregs, while the self-activated memory T cell CD127 is strongly expressed; therefore, CD4+CD25+CD127^ low^/- is used to represent Tregs now^4^, ^5^. Yu et al. reported that CD127 with low or no expression on the CD4+CD25+ marker is more specific than the combined markers CD39, CD73, and CD25+ for recognizing Tregs more accurately. The CD4+CD25+CD127-T cell population has the most typical characteristics of Tregs. At the same time, the expression level of the intracellular Foxp3 protein is also significantly higher than that in other cell populations. We conclude that simultaneous labeling of CD4+CD25+CD127^low^ on the cell surface is more accurate and more realistic for detecting Tregs than labeling of CD4+CD25+Foxp3.

The mechanism of action of CD4+CD25+Treg cells is unclear. It may be the secretion of immunosuppressive factors, such as transforming growth factor (TGF)-β (mainly TGF-β1), interleukin (IL)-10, and interferon (IFN)-γ into cytokines or on the cell surface. It plays an immunomodulatory role, and TGF-β promotes the development of regulatory T cells, maintains the expression of Foxp3 in CD4+CD25+Treg cells and, thus, regulatory T cells have a stable immunosuppressive function 6.

Tregs promote tumorigenesis and development by inhibiting the immune response of tumors. The role of Tregs in tumor immune escape is related to the secretion of inhibitory cytokines (mainly TGF-β and IL-10) by Tregs 7. IL-10 has an immunosuppressive function, and TGF-β directly inhibits activation of CD8+ cytotoxic T lymphocytes, significantly reducing the effect of tumor immunity^8^. TGF-β1 is a potent immunosuppressive factor that affects the proliferation, activation, and differentiation of innate and acquired immune cells and inhibits the production of immunoregulatory cytokines. IL-10 is an inhibitory cytokine produced by helper T2 cell subsets. Regulatory T cells produce immunosuppressive effects by secreting cytokines, such as IL-10, which inhibit inflammation and reduce the number of antigen-specific T cells 9-11.

To date, there is still no report on the clinical utility of CD4+CD25+CD127^Low^Tregs. The relationship between CD4+CD25+CD127^Low^Tregs and TGF-β1 and IL-10 levels remains unclear. Therefore, in this study, we observed the proportion of CD4+CD25+CD127^Low^Tregs in patients with primary hepatocellular carcinoma (HCC) before and after treatment and in healthy people, evaluate the clinical performance of CD4+CD25+CD127^Low^Tregs in patients at high risk of HCC. Then, the concentrations of TGF-β1 and IL-10 in patients with HCC before and after treatment were examined. The relationship between CD4+CD25+CD127^Low^Tregs and TGF-β1 and IL-10 levels, and the analysis of the differences in immune status of patients with liver cancer are expected to provide valuable and diagnostic reference data for clinical treatment, the disease course, and prognosis of liver cancer.

## Materials and Methods

### Clinical information

Sixty-nine patients (age, 31-68 years) confirmed by histopathological examination and diagnosed as the primary liver cancer were collected from the First Affiliated Hospital of Guangdong Pharmaceutical University. Patients were excluded if they had liver damage caused by drugs or ethanol and were treated with antiviral or immunosuppressive agents within 6 months. Peripheral blood of 87 healthy volunteers was collected as a control group. All liver functions, liver disease autoantibodies, routine blood scan, chest X-ray, abdominal B-ultrasound, electrocardiogram, and urine were normal.

This study was approved by the Institutional Ethics Review Board of the First Affiliated Hospital of Guangdong Pharmaceutical University and every subject signed a written informed consent.

### Reagents

The reagents were CD4-FITC, CD25-PE, mouse anti-human CD127-PerCP-CyTM 5.5, mouse IgG1-PE, mouse IgG1-PerCP-Cy5.5, hemolytic agent, and sheath fluid (BD Biosciences, San Diego, CA, USA). TGF-β1 and IL-10 kits were purchased from IBL (IBL International GmbH, Hamburg, Germany).

### Experimental method

**Figure 1:**
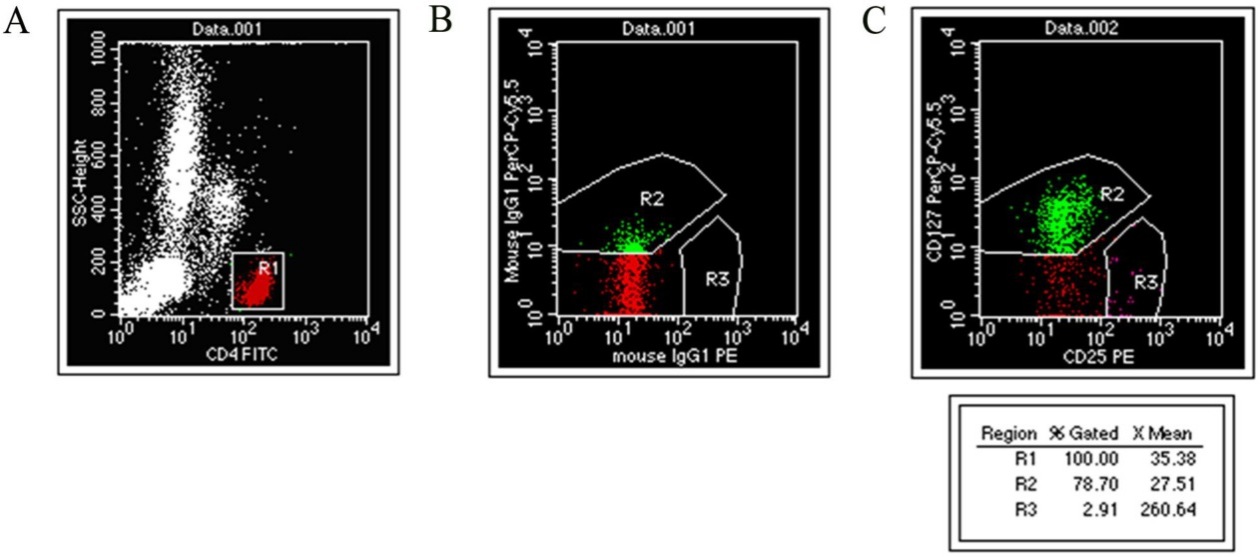
** Experimental analysis of CD4+CD25+CD127^low^ regulatory T cells.** (A) CD4+ / SSC scatter diagram. (B) Isotype Control. (C) CD4+CD25+CD127^low^ cell.

Two mL of venous blood was taken into EDTA-K3 coated tubes from fasting subjects in the morning. Each blood specimen was set up with two assay tubes and an isotype control. After adding 100 μL of anticoagulant to each tube, 10 μL of CD4-FITC, 10 μL of CD25-PE, PerCP-CyTM 5.5, and 2 μL of mouse anti-human CD127 were added to each assay tube. The same type of control was added to 10 μL of CD4-FITC and mouseIgG1-PE, along with 2.2 μL of mouseIgG1 percp-Cy5.5. The solutions were shaken and placed in the dark at room temperature for 15 min. One mL of hemolytic agent was added and vortexed in the dark at room temperature for 10 min. Then, the tube was centrifuged at 2,000 r/min for 2 min, the supernatant was discarded, 1 mL of sheath liquid was added and centrifuged at 2,000 r/min for 2 min, the supernatant was discarded, and 500 μL of sheath liquid was added, mixed well, and analyzed on the machine (Fig. 1).

### Consistency analysis of CD127 and FOXP3

CD4+CD25+CD127^Low^Tregs and CD4+CD25+Foxp3+Tregs were harvested from 10,000 cells/tube using CD4 gating. Cellquest software was applied to set the gate for CD4+FITC cells in the scatter diagram of the two CD4/SSC tubes, which is represented by a histogram and overlay histogram.

### Statistical Analysis

The statistical analysis was performed using SPSS18.0 software (SPSS Inc. Chicago, IL, USA). The data are expressed as mean ± standard deviation. The *t-*test of two sample means was used between the two groups. A paired *t*-test was used before and after treatment. One-way analysis of variance was used for multiple groups. A correlation analysis was conducted using Pearson's method. A P-value < 0.05 was considered significant. The area under the receiver-operating characteristic (ROC) curves (AUC) was quantified to evaluate the diagnostic performance of CD4+CD25+CD127^Low^Tregs ratio. The performance characteristics of CD4+CD25+CD127^Low^Tregs ratios were evaluated by determining the diagnostic sensitivity, specificity and diagnostic odds ratio (DOR) with 95% CIs.

## Results

### Clinical Characteristics of the Study Cohort

The clinical characteristics of patients with HCC were summarized in Table 1. When the subjects are younger than 49 years' old, the age between the HCC patients and the healthy people has no significant difference (p>0.05), the gender distribution has significant difference between HCC patients and the healthy people (p<0.01), the AFP levels in the HCC were significantly higher than the controls (p<0.01); When the subjects are older than 50 years' old, the age between the HCC patients and the healthy people has no significant difference (p>0.05), the gender distribution has significant difference between HCC patients and the healthy people (p<0.01), the AFP levels in the HCC were significantly higher than the controls (p < 0.01).

### Consistency between CD127 and Foxp3 expression

**Figure 2:**
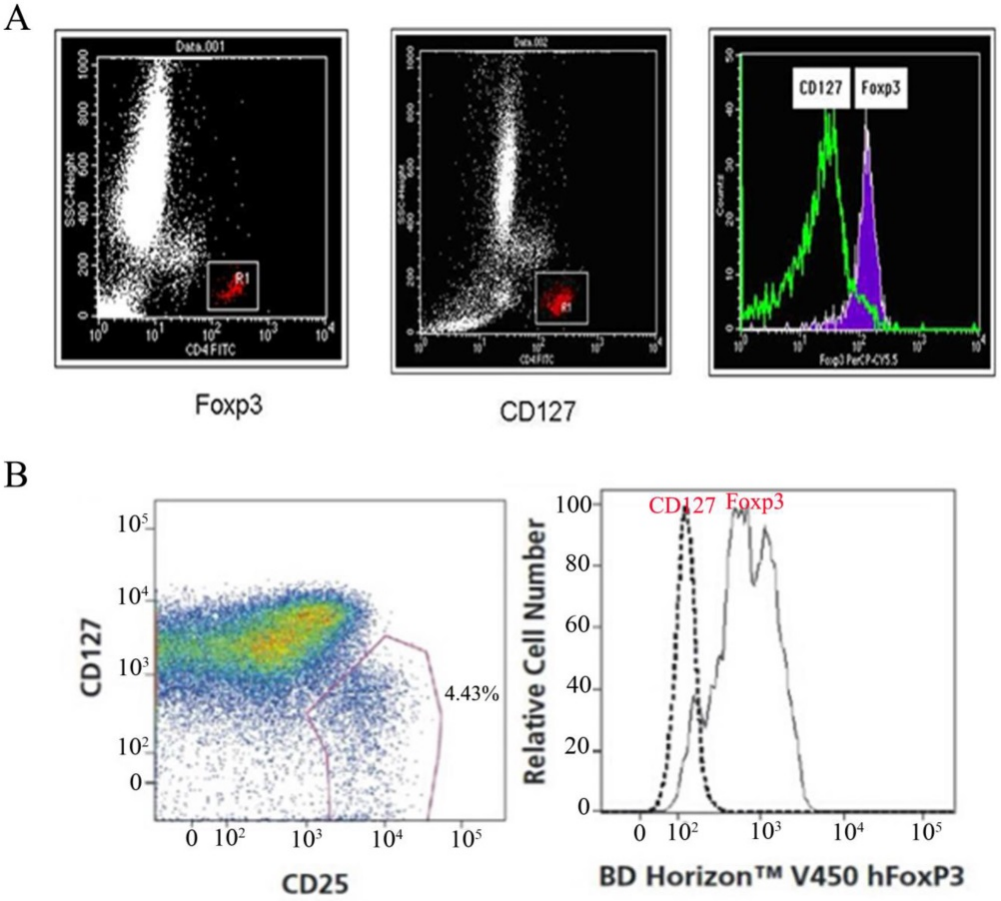
** Consistency of expression between CD127^low^ and Foxp3+ regulatory T cells.** (A) CD127+ and Foxp3+ regulatory T cells from one patient were investigated by FCS(BD FACS Calibur), consistency of expression between CD127^low^ and Foxp3+ regulatory T cells were analyzed by overlaying the two histograms.(B) CD127+ and Foxp3+ regulatory T cells from one patient were also investigated by FCS(FACSCantoTM II), consistency of expression between CD127^low^ and Foxp3+ Tregs were analyzed.

It has been reported that high expression of Foxp3 (Foxp3^high^) and low expression of CD127 (CD127^low^) may originate from the same group of cells 12. CD4+CD25+Foxp3 and CD4+CD25+CD127 were used as phenotype markers to identify regulatory T cells in normal human peripheral blood whole blood specimens. The consistency analysis of CD127 and Foxp3 cells by BD FACS Calibur flow cytometry showed that the two groups of cells were almost in the same region (Fig. 2).

### Comparison of CD4+CD25+CD127^low^Tregs in the peripheral blood of the primary liver cancer and healthy control groups

**Figure 3:**
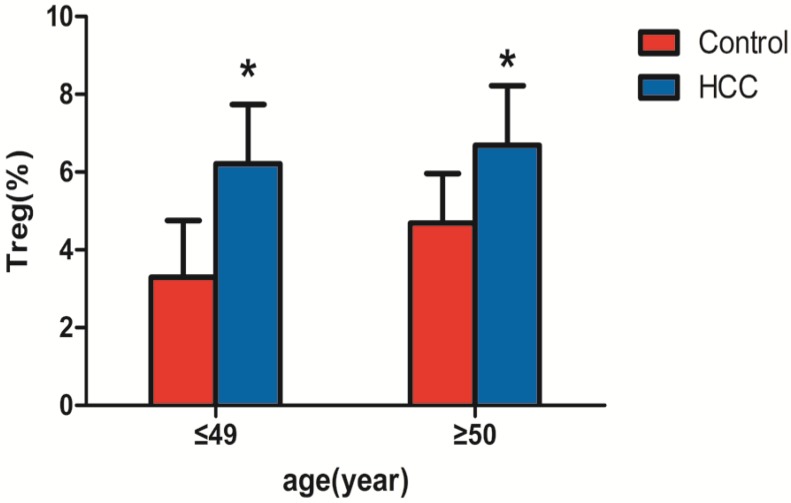
** Comparison of CD4+CD25+CD127^low^ regulatory T cells in peripheral blood of patients with liver cancer and the healthy control group.** Note: * indicates that patients with liver cancer ≤ 49 years old (n=37) were significantly different compared with the healthy control group (n=45) (P < 0.05), and patients with liver cancer ≥ 50 years old (n=32) were significantly different compared with the healthy control group (n=42) (P < 0.05).

Among the 69 patients with liver cancer, 37 patients were ≤ 49 years old and 32 patients were ≥50 years old. Significant differences were observed between the two groups by the independent samples *t-*test (all* P < 0.05*). As shown in Table 2 and Figure 3, CD4+CD25+CD127^low^Tregs in peripheral blood of patients with liver cancer in those≤ 49 years old (6.21±1.53%) were significantly higher than those in health subjects with the same age (3.30±1.45%; P<0.05). CD4+CD25+CD127^low^Tregs in peripheral blood of patients with liver cancer in those≥50 years old (6.69±1.53%) were significantly higher than those in health subjects with the same age (4.69±1.27%; P<0.05).

### Changes in CD4+CD25+CD127^low^Tregs before and after treatment of liver cancer

**Figure 4:**
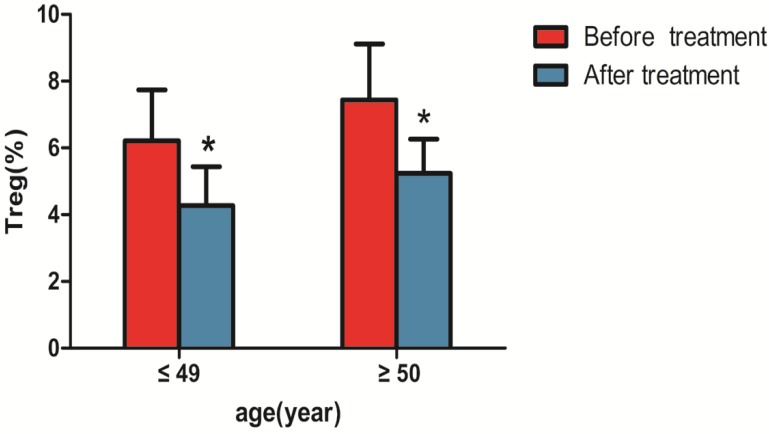
** Comparison of CD4+CD25+CD127^low^ regulatory T cells in peripheral blood of patients with liver cancer before and after treatment.** Note: * indicates that with liver cancer patients ≤ 49 years old after treatment were significantly different compared with the patients before treatment (n=37, P < 0.05) , and patients with liver cancer ≥ 50 years old after treatment were significantly different compared with the patients before treatment (n=32, P < 0.05).

Changes in CD4+CD25+CD127^low^Tregs were observed before and after 1 month of treatment., as shown in Table 3 and Figure 4, the percentage of CD4+CD25+CD127^low^Tregs in patients aged ≤ 49 years (6.21±1.53%) before treatment were higher than that after treatment (4.27±1.17%; P<0.05). the percentage of CD4+CD25+CD127^low^Tregs in patients aged ≥50 years (7.44 ± 1.67%) before treatment were higher than that after treatment (5.24 ± 1.02%; P<0.05).

### Clinical Performance Evaluation of CD4+CD25+CD127^low^Tregs

**Figure 5:**
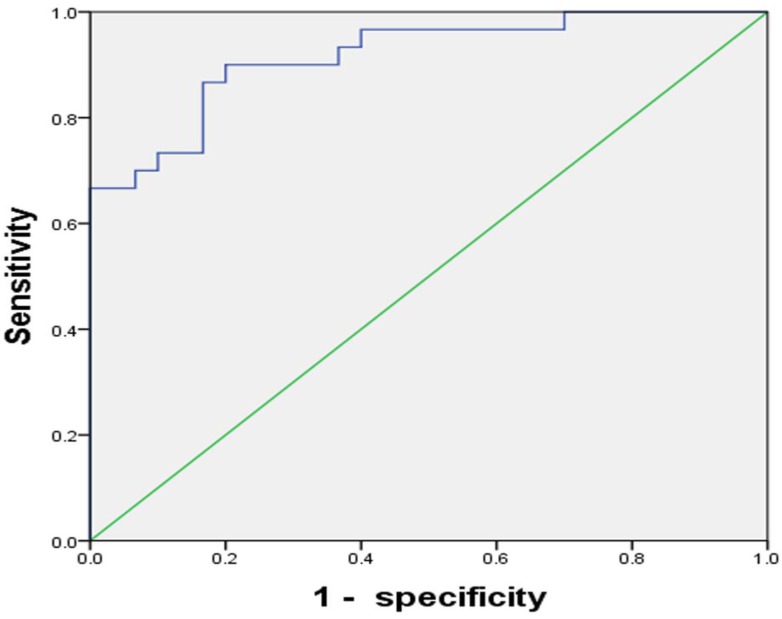
** The ROC curve of CD4+CD25+CD127^low^Tregs ratios whose AUC was 0.917.** The best sensitivity and specificity at the best cutoff point were 90% and 80% respectively.

To test the clinical utility of CD4+CD25+CD127^low^Tregs in patients with HCC, the ROC curve was analyzed. The results showed the AUC for CD4+CD25+CD127^low^Tregs ratio was 0.917(95% CI, 0.848-0.986), with a diagnostic sensitivity of 90%, specificity of 80% (Figure 5).

### Comparison of TGF-β1 and IL-10 concentrations before and after treatment in patients with primary liver cancer

As shown in Table 4, the TGF-β1 concentration before treatment of primary liver cancer (1,260.80± 359.87 ng/L) was higher than that after treatment (896.96± 287.25 ng/L; P < 0.05). The IL-10 concentration before treatment of primary liver cancer (577.49 ± 161.75 ng/L) was higher than that after treatment (480.43 ± 177.79 ng/L; P < 0.05).

### Correlation between the percentage of CD4+CD25+CD127^low^Tregs and the concentrations of TGF-β1 and IL-10

The relationships between the percentage of CD4+CD25+CD127^low^Tregs and the concentrations of TGF-β1 and IL-10 were compared. Pearson's method was used for the correlation analysis.

**Figure 6:**
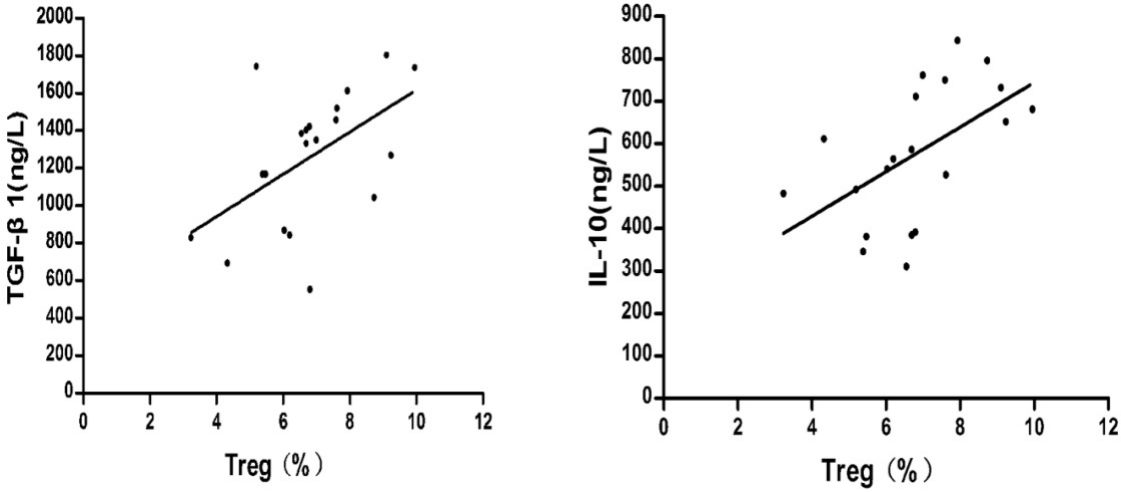
Correlation between the percentage of regulatory T cells and the concentrations of TGF-β1 and IL-10 before treatment of liver cancer.

As shown in Fig.6, before liver cancer treatment, the TGF-β1 and IL-10 concentrations were positively correlated with the percentage of CD4+CD25+ CD127^low^Tregs in patients with HCC respectively ((r = 0.526, P = 0.017; r = 0.546, P = 0.013).

**Figure 7:**
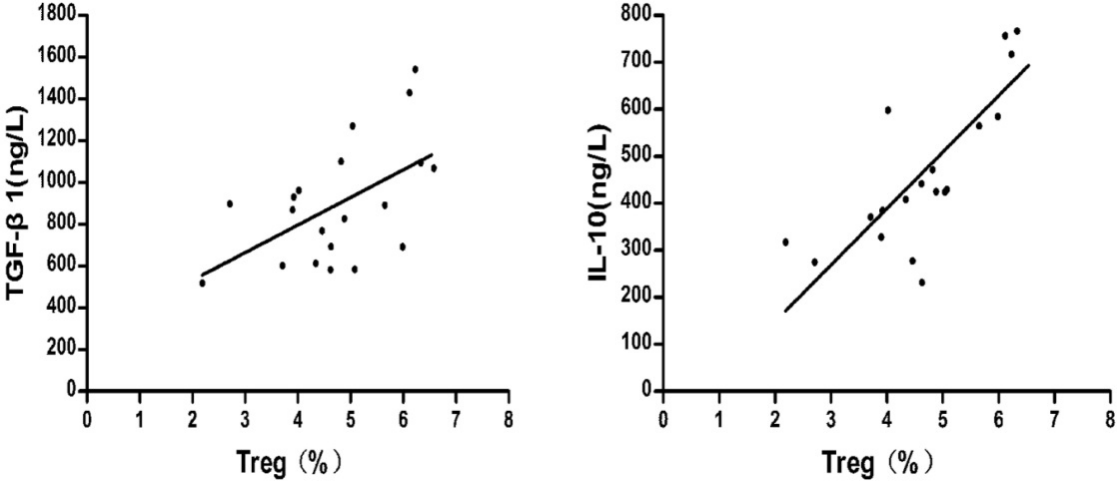
Correlation between the percentage of regulatory T cells and the concentrations of TGF-β1 and IL-10 after treatment of liver cancer.

As shown in Fig.7, after liver cancer treatment, the TGF-β1 and IL-10 concentrations were positively correlated with the percentage of CD4+CD25+ CD127^low^Tregs in patients with HCC respectively (r = 0.543, P = 0.013; r = 0.789, P=0.000).

## Discussion

Tregs inhibit the host's anti-tumor immune response, and have a clear correlation with the disease process. CD4+CD25+Tregs can be detected in peripheral blood of patients with gastric cancer, lung cancer, ovarian cancer, liver cancer, pancreatic cancer and breast cancer, local tumors, invasive lymph nodes, and drainage lymph nodes, and the number of CD4+CD25+Treg cells is negatively correlated with the disease course and the prognosis; the higher the number of Tregs, the worse the prognosis 13.

Liver cancer is a common tumor, as it has the third highest incidence in China, and the mortality rate is second among malignant tumors. The prognosis for liver cancer is poor. Tregs are a subset of CD4+ T cells with immunoregulatory functions that inhibit the activation and proliferation of anti-tumor effector cells and are associated with tumor immune escape^14^. Ormandy^15^ et al. reported that the number of Tregs in peripheral blood of patients with liver cancer increases significantly. The number of Tregs in patients with liver cancer may be a prognostic indicator of HCC 16. However, other studies have shown that the percentage of regulatory T cells in peripheral blood of patients with primary liver cancer is not different from that of a healthy control group 17. Differences in the criteria for enrolling patients, different methods for detecting Tregs, and different cases are possible reasons for this difference.

At present, the technology for detecting Tregs by flow cytometry is insufficient. The main problem is the lack of specific markers. Previous studies on Tregs have mainly focused on Foxp3 in CD4+CD25+ cells. Foxp3 is considered a key marker molecule specifically expressed in CD4+CD25 +Tregs, which is closely related to cell differentiation, development, and functional maturation 18. However, as Foxp3 is intracellular expressed, it is necessary to rupture the membrane during detection and analysis; the detection process will affect cell activity, thus affecting the accuracy of the test results 19. Therefore, to study the role of Tregs in tumor diseases more effectively, it is necessary to find a more sensitive Tregs surface marker.

The CD127 molecule on the surface of the CD4+CD25+ cell population is also a specific marker for this group of cells. A good correlation has been observed between high expression of Foxp3 and low expression of CD127 in this group of cells; that is, high expression of Foxp3 (Foxp3^high^) and low expression of CD127 (CD127^low^) may be the same group of cells 12. CD127 is mainly expressed on the surface of mature T cells in normal human peripheral blood, and *in vitro* experiments show that regulatory T cells with inhibitory activity express low levels of CD127 on the surface. Therefore, CD4+CD25+CD127^Low^ is currently considered to be a more valuable molecular surface marker to detect regulatory T cells more specifically than CD4+CD25+Foxp3+. Our study showed that CD4+CD25+Foxp3 and CD4+CD25+CD127^Low^ were phenotypic markers for identifying regulatory T cells in the same normal human peripheral blood sample. The two groups of cells are roughly in the same region, as shown in Figure 2. Foxp3 and CD127 are expressed by regulatory T cells.

In this study, the proportion of peripheral blood CD4+CD25+CD127^low^Tregs accounted for CD4+ T lymphocytes in patients with primary liver cancer (6.21 ± 1.53% for ≤ 49 years and 6.69 ± 1.53% for ≥ 50 years) and healthy control groups (3.30 ± 1.45% for ≤ 49 years and 4.69 ± 1.27% for ≥ 50 years) (P <0.05). These results suggest that the number of CD4+CD25+CD127^low^ Treg regulatory T cells in the peripheral blood of patients with liver cancer is significantly higher than that of the control group. Regulatory T cells declined in patients with liver cancer treated for 1 month. Because the subjects of this study were patients with primary liver cancer who were initially diagnosed and not treated clinically, the effects of an immunomodulator and other factors, such as chemotherapy, were excluded. The results suggest that CD4+CD25+CD127^low^Tregs are closely related to the occurrence and development of tumors.

Though the role of CD4+CD25+CD127^low^Tregs on the cancers has been reported, its clinical utility has not been fully elucidated. In the present study, we retrospectively investigated the clinical performance of CD4+CD25+CD127^low^Tregs in the population undergoing initial or repeated biopsy and at high risk of HCC. The results showed the AUC for CD4+CD25+CD127^low^Tregs ratio was 0.917(95% CI, 0.848-0.986), with a diagnostic sensitivity of 90%, specificity of 80% (Figure 5). To our best knowledge, it is the first report on the clinical utility of CD4+CD25+CD127^low^Tregs.

The mechanism of action of Tregs may be that of secreted inhibitors, such as the cytokines TGF-β1, IL-10, and IFN-γ 20, which play an immunomodulatory role. CD127 is the IL-7 receptor alpha chain, and the specific response of T lymphocytes to IL-7 is mainly achieved through CD127. Studies have confirmed that the expression of TGF-β1 is closely related to the proliferation and differentiation of CD4+CD25+Tregs 21. Overexpression of TGF-β1 in peripheral lymphoid organs increases peripheral Treg and Foxp3 expression, and blocking the T cell TGF-β1 signaling pathway produces the opposite effect, indicating that TGF-β1 plays an important role in regulating the peripheral blood CD4+CD25+ T cell pool and Foxp3 expression 22.

In a study of ulcerative colitis, CD4+CD25+ Tregs in rat peripheral blood were negatively correlated with IL-10 levels 23. The level of CD4+CD25+CD127-Tregs in peripheral blood of patients with lung cancer is positively correlated with plasma IL-10 level 24. CD4+CD25+ Foxp3+ Tregs are positively correlated with changes in IL-10 and TGF-β1 in patients with rheumatoid heart disease 25. In the study of children with juvenile idiopathic arthritis (JIA), serum levels of IL-10 and TGF-β1 did not correlate with the proportion of regulatory T cells (CD4+CD25highFOXP3+) in peripheral blood from JIA patients and healthy controls 26.

The correlation between regulatory T cells and TGF-β1 and IL-10 has been widely reported, and the results of this study show that CD4+CD25+CD127^low^ Tregs were positively correlated with TGF-β1 and IL-10 before and after treatment of liver cancer. These findings are consistent with other reports of TGF-β1 and IL-10 25. However, he specific mechanism is still unclear and further research is needed.

In present study, the proportion of CD4+CD25+CD127^low^Tregs was positively correlated with TGF-β1 and IL-10 concentrations, which was consistent with relevant reports. This study suggests that CD4+CD25+CD127^low^Tregs, TGF-β1, IL-10 are closely related to the occurrence and development of tumor. The increase of Tregs in patients with liver cancer may be related to immune escape by the liver cancer. This study used CD4+CD25+CD127^low^ as a marker, consistent with the report of CD4+CD25+ Foxp3+-tagged regulatory T cells in liver cancer, which was higher than that of healthy control^27^. However, the production of CD4+CD25+CD127^low^ Tregs and its related mechanisms in tumorigenesis remain unknown. Therefore, the correlation between the percentage of CD4+CD25+CD127^low^Tregs and the concentrations of TGF-β1 and IL-10 in this study will provide some insight for more in-depth studies on the tumorigenetic mechanism, and facilitate the development of more effective tumor treatment programs.

## Figures and Tables

**Table 1:** Clinical Characteristics of Study Population

		≤ 49 years old				≥ 50 years old		
		Control	Liver cancer	p		control	Liver cancer	p
**Age**		41.29+7.96	42.12+5.82	>0.05		61.06+8.20	60.25+6.65	>0.05
**Sex**	Male	23	30			22	26	
	Female	22	7	<0.01		20	6	<0.01
**AFP(ng/mL)**		6.74±1.52	907.83±1172.70	<0.01		7.79±2.95	1073.32±1565.13	<0.01

**Table 2:** Comparison of CD4^+^CD25^+^CD127^low^ regulatory T cells in peripheral blood of liver cancer patients and healthy control group.

Group	N	CD4+CD25+CD127^low^ regulatory T cells (%, x±s)
control group (aged ≤ 49 years)	45	3.30±1.45
Liver cancer group (aged ≤ 49 years)	37	6.21±1.53*
control group (aged ≥50 years)	42	4.69±1.27
Liver cancer group (aged ≥50 years)	32	6.69±1.53*

Note: * indicates that the difference between the liver cancer group and the normal group at the same age is statistically significant (P<0.05)

**Table 3:** Comparison of CD4^+^CD25^+^CD127^low^ regulatory T cells in peripheral blood of liver cancer patients before and after treatment(%, x±s).

Group	CD4+CD25+CD127^low^ regulatory T cells (%, x±s)
before treatment (aged ≤ 49 years)	6.21±1.53
after treatment (aged ≤ 49 years)	4.27±1.17*
before treatment (aged ≥50 years)	7.44±1.67
after treatment (aged ≥50 years)	5.24±1.02*

Note: * indicates statistically significant difference before and after treatment in the same age group (P<0.05)

**Table 4:** Comparison of TGF-β1 and IL-10 concentrations before and after treatment of liver cancer

group	TGF-β1(ng/L)	IL-10(ng/L)
Before treatment	1260.80±359.87	577.49±161.74
After treatment	896.96±287.25*	480.43±177.79**

Note: * indicates that TGF-β1 is statistically significant before and after treatment (P<0.05); **IL-10 is statistically significant before and after treatment (P<0.01)
